# Examining the impact of total sleep duration on daily affect among short-sleeping adolescents: an experimental study

**DOI:** 10.1093/abm/kaag008

**Published:** 2026-03-29

**Authors:** Avah Mousavi-Raad, Ariel Neikrug, Dean Beebe, Uma Rao, Jessica Borelli, Kate R Kuhlman

**Affiliations:** RAND Corporation, Division of Social and Economic Well-being, Santa Monica, CA 90401, United States; Department of Psychiatry & Human Behavior, School of Medicine, University of California Irvine, Irvine, CA 92617, United States; Division of Behavioral Medicine and Clinical Psychology, Cincinnati Children’s Hospital Medical Center, Cincinnati, OH 45229, United States; Department of Pediatrics, University of Cincinnati College of Medicine, Cincinnati, OH 45229, United States; Department of Psychiatry & Human Behavior, School of Medicine, University of California Irvine, Irvine, CA 92617, United States; Department of Psychological Science, School of Social Ecology, University of California, Irvine, Irvine, CA 92617, United States; Department of Psychiatry, Children’s Hospital of Orange County (CHOC), Orange, CA 92868, United States; Cousins Center for Psychoneuroimmunology, Semel Institute for Neuroscience & Human Behavior, University of California, Los Angeles, Los Angeles, CA 90095, United States; Department of Psychological Science, School of Social Ecology, University of California, Irvine, Irvine, CA 92617, United States; Department of Psychological Science, School of Social Ecology, University of California, Irvine, Irvine, CA 92617, United States; Department of Psychiatry, Children’s Hospital of Orange County (CHOC), Orange, CA 92868, United States; Cousins Center for Psychoneuroimmunology, Semel Institute for Neuroscience & Human Behavior, University of California, Los Angeles, Los Angeles, CA 90095, United States

**Keywords:** adolescents, actigraphy, daily diary, mood, sleep extension, emotional well-being

## Abstract

**Purpose:**

This study examined whether increasing sleep duration was associated with changes in daily affect, specifically, increases in positive affect (PA) and decreases in negative affect (NA), among short-sleeping adolescents.

**Methods:**

Healthy, short-sleeping adolescents (reporting <8 h sleep on school nights based on parent and adolescent screening questionnaires, *n* = 41, 14-17 years) participated in 1 week of baseline monitoring, then were randomized to either sleep extension (EXT; +90 min in bed; *n* = 21) or habitual sleep (HAB; *n* = 20) for 2 weeks. Both conditions established fixed bedtime and rise-time to reduce sleep variability. Sleep duration was assessed via wrist actigraphy. Participants completed the 22-item Profile of Mood States twice daily: soon after waking and at bedtime during the 3-week protocol.

**Results:**

During the initial preintervention week, average nightly sleep duration was 6.42 h (SD = 1.16). In the experimental weeks, HAB averaged 6.22 h (SD = 0.95), while EXT increased to 7.00 h (SD = 0.68; *P* = .002). This between-group difference represented a large effect (Hedges’ *g* ≈ 0.87), confirming that the manipulation increased sleep duration. Morning PA increased significantly in both conditions (*P* < .001). There were no significant differences in morning or evening affect between the EXT and HAB conditions. Adolescents experienced lower morning NA after nights when they slept longer, based on a secondary analysis conducted in the full sample, regardless of the experimental condition (*P* = .005). Further, adolescents with higher average sleep duration had lower evening PA regardless of the experimental condition (*P =* .03). Morning PA increased from baseline to the experimental weeks in both conditions. Unexpectedly, higher between-person total sleep duration was associated with diminished evening PA, emphasizing the nuanced role of sleep patterns in shaping affective states.

**Conclusions:**

Increasing nightly sleep is feasible for short-sleeping adolescents and resulted in longer sleep duration. Although affect did not differ between groups over the short intervention period, daily associations suggest that sleep may play a role in adolescents’ emotional experiences. Longer or more intensive sleep interventions may be needed to detect group-level changes in affect.

## Introduction

Nocturnal sleep plays an important role in adolescents’ health because their biological systems are still developing. Obtaining the recommended 8-10 h of nightly sleep has been linked to multiple domains of functioning (ie, academic achievement, emotion regulation) and physical and mental health among adolescents.^1–3^ However, about half of the adolescents in the United States regularly sleep less than 8 h on school nights, placing them in the short-sleeping range.^4^ Such chronic short sleep could have cumulative, long-lasting, and adverse consequences,^5^ including increased risk of mental and physical problems later in life.^6^ Even so, most relevant findings have been correlational, with limited data to speak to whether lengthening adolescent sleep can mitigate health risks. The complexity of this issue is compounded by variability in sleep patterns, leaving the question of which factor (ie, sleep duration or variability) holds greater significance unanswered.

As highlighted in other studies,^7^^,^^8^ adolescent sleep interventions have rarely examined mechanisms of change which are essential for identifying intervention targets that have the potential to mitigate health disparities.^9^^,^^10^ Correlational studies have observed short sleep to be linked with greater negative affect (NA), such as feelings of anger and anxiety,^11–13^ and less positive affect (PA), such as decreased happiness compared to adolescents who get the recommended amount of sleep.^11^^,^^12^^,^^14^ Variable and irregular sleep patterns have also been linked to affect during adolescence, such as decreased positive mood.^15^

Importantly, prior research has relied on trait-like assessments of affect, often measured over weeks or months, which may fail to capture day-to-day variability in affective states. For example, 4 randomized studies^12^^,^^16–18^ found that sleep restriction heightens negative emotions and lowers positive emotions,^12^^,^^16–19^ and 1 study found that extending the sleep of habitually short-sleeping adolescents led to less NA and increased PA.^20^ However, in each case the measures of affect occurred at 1-2-week intervals. This precludes a more comprehensive examination of the links between daily changes in behavioral factors such as sleep and changes in affect.^21^ Recent studies show that daily affect ratings can provide a more nuanced assessment of affect dynamics in real-life settings.^21^

### Present study

**Figure 1:**
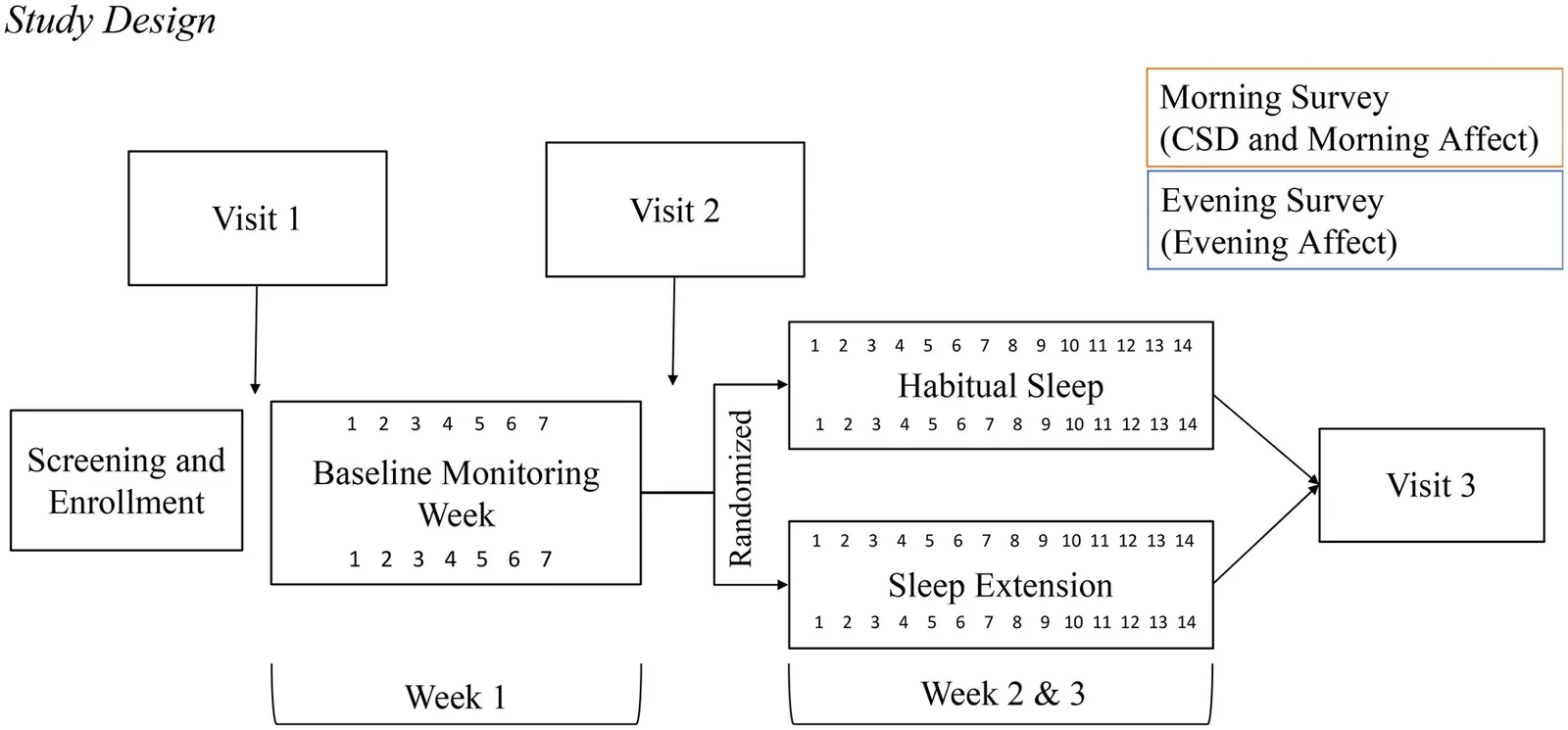
Study design.

Building off an established experimental sleep extension (EXT) protocol^20^ in a new sample, this study integrated daily measures of sleep and affect, aiming to assess whether increasing total sleep duration might contribute to improving daily affect, specifically reducing NA and increasing PA in adolescents experiencing insufficient sleep. Adolescents (14-17 years) participated in 1 week of baseline monitoring sleep, and then short-sleeping adolescents (ie, those who slept less than 8 h) were randomized to either an sleep EXT protocol (extending their time in bed by 90 min) or an HAB sleep condition (continued short-sleeping) for 2 weeks (see Figure 1). Specifically, in both conditions, the experimental weeks involved establishing fixed bed- and rise-times to reduce sleep variability and therefore isolate sleep duration. We examined whether changes in affect from baseline to the experimental weeks differed between adolescents in the HAB and EXT conditions. This study also had a secondary aim to assess whether adolescents experienced more PA and less NA on days after they slept longer (daily associations, using each person as their own control), as well as whether adolescents with higher average sleep had a higher PA and lower NA on average (between-person association), regardless of experimental condition. We hypothesized that, on days when adolescents sleep longer, they would experience lower NA and greater PA, as well as that adolescents with higher average total sleep duration would have lower NA and greater PA. These analyses were planned as secondary aims, to complement the primary condition-based hypothesis by evaluating within- and between-person sleep-affect dynamics across the full sample.

## Methods

### Participants

**Figure 2:**
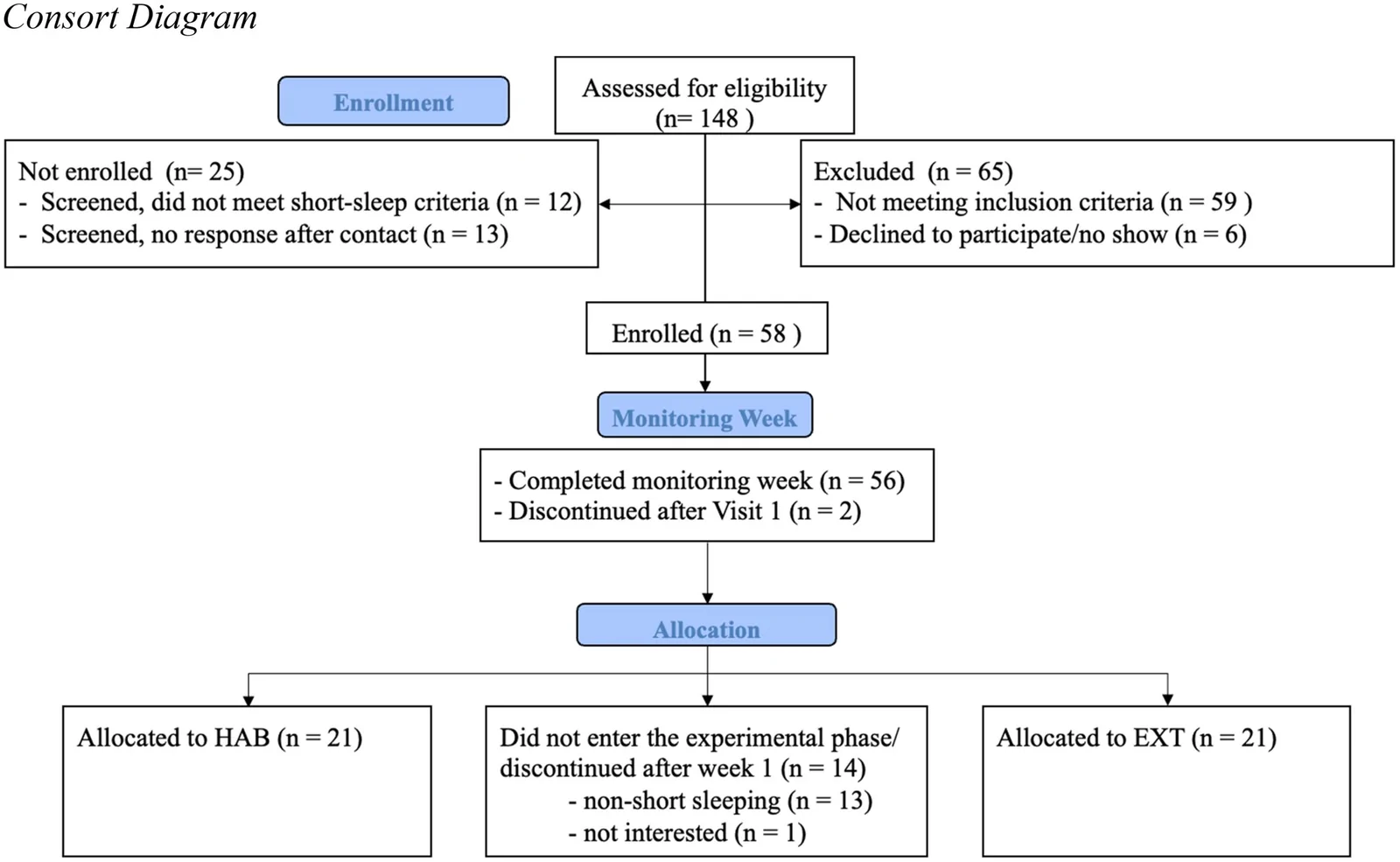
Consort diagram.

The sample included 41 adolescents (*n* = 21 EXT and *n* = 20 HAB) residing in Orange County, CA who completed a baseline assessment between April 2022 and May 2023. Eligible participants were adolescents aged 14-17 years at baseline who averaged 5-7 h of sleep per night on weekdays. Adolescents were eligible if they regularly obtained <8 h of sleep on school nights based on parent and adolescent screening questionnaires.^4^^,^^20^ This short-sleep criterion was confirmed using daily self-reported sleep times during the baseline monitoring week. Adolescents with a current medical or psychiatric condition (per parent-report), who were taking medications known to influence their sleep or daytime alertness, who had a history of neurological illness or injury, were diagnosed with an intellectual disability, or had a diagnosis of obstructive sleep apnea were excluded. In accordance with the study’s primary objective to recruit short-sleeping adolescents, adolescents with shorter sleep durations (based on parental reports) were given enrollment priority. It is important to note that individuals who were initially part of the monitoring week but were found to not meet the criteria for short-sleeping (ie, those who slept at least 8 h) were not included in the final dataset. Of the 58 adolescents who initially enrolled in the study, 2 withdrew before the monitoring week, 1 before randomization, and 13 were excluded/dropped (due to not being short-sleeping based on self-reports in the baseline week) (Figure 2) and 1 participant’s objective sleep monitor (Actiwatch) did not collect data due to malfunction and therefore were excluded from the study.

### Procedures

This randomized sleep EXT study was conducted specifically to examine the impact of increasing sleep duration on daily affect in adolescents. All study procedures were approved by the UC Irvine Institutional Review Board (UCI #340). Data were collected between March 2022 and May 2023. Participants were recruited through mass mailings to households likely to have children aged 14-17 years (2015 census data) and via the UCI Child Studies Collaborative, which uses Orange County birth records to identify families interested in pediatric research (https://www.uciscienceoflearning.org/child-studies-collaborative.html). Families received flyers and, if interested, contacted the lab for details and a 15-min eligibility phone screen. Enrollment was scheduled to avoid the first and last weeks of a semester or multiday school breaks; summer enrollment was limited to those in structured activities (eg, summer school); 4 participants met this criterion.

The study included 3 sessions with participants and their families, along with a 3-week period of home-based longitudinal data collection (see Figure 1). At visit 1, participants and parents provided assent or consent, completed demographic and psychosocial questionnaires via REDCap,^22^ and reported typical rise-times and bedtimes. Next, participants were given an actigraph (detailed below) and trained to use it, and instructed to wear it on the nondominant wrist at all times, except when in ocean water.

For the 3-week at-home period, participants received daily text messages at their individual rise-time and 1 h before bedtime. Each message included the next night’s sleep instructions and a link to the daily diary. Morning diaries assessed the prior night’s sleep and current affective state; evening diaries assessed current affect, napping, caffeine intake, and exercise that day. Participants were encouraged not to nap and to limit caffeine to 1 coffee or energy drink (∼95 mg) or 2 caffeinated sodas (∼80 mg) per day.

#### Baseline monitoring week (week 1)

Throughout the study, adolescents slept in their normal home setting, with sleep monitored via daily diaries and actigraphy. In week 1, they maintained their typical self-reported bed- and rise-times. At week’s end, self-reported sleep data were reviewed to confirm eligibility (averaging <8 h per night).

#### Experimental weeks (weeks 2 and 3)

Participants were randomized prior to enrollment using a 1:1 allocation in blocks of 10. Self-reported bed- and rise-times from week 1 determined the schedule for the experimental weeks. After reviewing baseline sleep data at visit 2, the random assignment was revealed (HAB or EXT). For HAB, the researcher problem-solved ways to maintain the baseline schedule, including reviewing daily routines, discussing healthy sleep habits, addressing barriers, and developing strategies for a consistent bedtime and wake-up, including weekends. For EXT, the researcher problem-solved ways to add 90 min in bed, reviewing schedules, discussing healthy sleep, addressing barriers, and identifying where and how to extend time in bed.

During weeks 2 and 3, HAB participants maintained their baseline sleep schedule (averaged across night-to-night variability^20^), while EXT participants adjusted bedtimes to add 1.5 h in bed relative to week 1. Nonadherence to bedtime or rise-time (within 30 min) triggered a text after 1 day or a call after 2 days. In this phone call, research assistants emphasized the importance of adherence to the protocol. Adolescents were instructed to have lights and electronics off by bedtime for 2 weeks, with parental support. Participants also were asked to maintain their sleep schedule during the weekend, awakening no more than an hour later and having a similar bedtime as weeknights.^20^

At visit 3, marking the end of the 2-week experimental phase, participants returned the actigraph and completed online demographic and psychosocial questionnaires (eg, affect and covariates). Adolescents were also compensated in cash up to $250 and incentivized for completing all study procedures. Specifically, participants were compensated $20 for each laboratory visit (3 visits in total), $8 for the completion of each day of daily diaries (21 days in total) and received a $22 bonus at the end of the study if they had a response rate above 80% and for returning the actigraph. Participants who withdrew were compensated proportionally for completed components. Additional procedural details regarding intervention delivery are available from the authors upon request.

### Measures

#### Sleep

We used actigraphs to measure sleep duration. Actigraphs are lightweight and wearable devices designed to record movement and activity over time. Compared to overnight polysomnography, actigraphs have as high as 97% sensitivity for detection of wakefulness during sleep.^23^ In this study, participants wore the Respironics Actiwatch Spectrum Plus, a lightweight, water-proof wrist-worn accelerometer for the total duration of the study (21 days). To score sleep and waketime, the actigraphs were programmed to record in 60-s epochs at a medium sensitivity level. At the end of the data collection period, data were downloaded onto a desktop computer.

We used sleep diaries to identify the windows of time in bed, including bedtime and rise-time. In cases where sleep diaries were missing, we utilized actigraphy data to determine these windows. Then, *sleep duration* was computed using the Actiware program algorithms based on the rest intervals, defined as the difference between sleep onset and offset, subtracting nocturnal awakenings. Our study demonstrated a 95.1% completion rate for actigraph data across 42 participants, which resulted in 839 data points; 1 participant’s actigraph did not collect data during the experimental weeks due to malfunction and therefore was excluded from the study (*n* = 41). Although not included in primary analyses, for descriptive purposes we also recorded sleep efficiency/continuity, which is the ratio of time sleeping to time in bed, accounting for sleep onset latency and wake after sleep onset.

In addition to actigraphy-based sleep duration, participants also provided self-reported sleep duration through the 9-item Consensus Sleep Diary (CSD),^24^ completed each morning. The CSD includes an item asking, “In total, how long did you sleep?” which was used to capture subjective sleep duration for descriptive purposes. Subjective sleep quality was similarly collected for descriptive purposes via the CSD, where participants reported their quality of sleep the prior night on a 5-point Likert scale (1 = “very poor”, 5 = “very good”).

#### Affect

Participants completed the 22-item Profile of Mood States (POMS)^25^ 2 times per day: every morning after waking up, and at bedtime. Participants were asked to indicate the degree to which they experienced each state that day on a 5-point Likert scale (1 = *not at all* to 5 = *extremely*). Across the 41 participants, we had a 97.4% completion rate for morning daily diaries, resulting in 839 complete diaries, and a 96.1% completion rate for evening daily diaries, resulting in 828 complete diaries.

*Positive affect* was computed using 3 items reflecting the vigor subscale (experiencing vigor, cheerfulness, liveliness), and 4 additional low-arousal PA items that have been used in previous daily diary studies focusing on the role of biobehavioral factors in affect.^26^ These included enthusiastic, interested, content, and grateful.^27^^,^^28^ These additional items broaden the expression of PA beyond high-arousal states to capture a wider range of daily positive emotional experiences that are relevant in this population. This adapted approach has been used in prior work examining daily affective fluctuations in adolescents.^26–28^ For morning PA, between-person reliability (*R_kf_*) was 0.98, indicating excellent reliability in capturing stable differences between individuals, and within-person change reliability (*R_c_*) was 0.89, suggesting excellent reliability for detecting fluctuations over time. For evening PA, *R_kf_* was 0.97, indicating excellent between-person reliability, and *R_c_* was 0.81, reflecting excellent within-person change reliability.

*Negative affect* was computed using 9 items, which reflected scores on depressed mood (feeling sad, hopeless, discouraged), tension (feeling anxious, uneasy, on edge), and anger (experiencing anger, resentment, annoyance) subdomains.^25^ The original POMS Negative Affect subscale includes a broad range of negative emotional states that map well onto daily experiences, so the full item set was retained for NA without adaptation. For morning NA, *R_kf_* was 0.95, indicating excellent reliability in capturing stable differences, and *R_c_* was 0.56, suggesting moderate reliability for detecting changes over time. For evening NA, *R_kf_* was 0.95, reflecting excellent between-person reliability, and *R_c_* was 0.59, indicating moderate within-person change reliability.

### Data analysis

#### Preliminary analyses

All data analysis was conducted in SPSS software version 29.0. All data were assessed for normality, and heteroscedasticity and found to fulfill the assumptions necessary for parametric statistical analyses. We examined distributions for outliers. A small number of unusually long sleep durations (eg, >10 h) occurred, typically on weekend mornings. These values were retained because they reflected valid sleep periods (eg, catching up after school nights). Descriptive statistics (eg, means and SDs, frequencies, and histograms) were computed for all variables, along with 95% bootstrap CIs. Statistical significance was defined as *P* <.05 or a 95% CI that excluded zero.

Preliminary analyses ensured comparability between adolescents randomized to the EXT and HAB conditions on demographic characteristics and primary variables using *t*-tests. We also examined associations between potential covariates and our outcomes. If there was a significant association between potential covariates and outcomes, we included them in the models as a covariate. These variables included sex^29^ and age.^30^ Potential covariates included daily napping (yes/no), caffeine consumption (yes/no), exercise (yes/no), and school vs nonschool day (0 = nonschool day, 1 = school day). Sex was a significant predictor of both morning and evening NA, with females reporting higher NA. Additionally, exercise (yes/no) was a significant predictor of evening PA, with exercise associated with higher PA. As a result, these variables were included in the corresponding analyses.

Preliminary analyses also tested for changes in sleep from the baseline week to the experimental weeks within each experimental condition via paired sample *t*-tests to determine the effectiveness of the manipulation. Further, a validation check was conducted to ensure that sleep duration in the EXT condition was longer on average across the 2 weeks relative to the HAB condition.^20^

#### Primary analyses

We examined our hypotheses by employing linear mixed models with the restricted maximum likelihood estimation (REML) approach. We performed within- and between-person analyses centering on sleep duration to yield orthogonal within- and between-person versions of these predictors.^31^ Specifically, daily and person-level variations in daily sleep duration were separated by disaggregating the predictor (sleep duration) each day *i* for person j (ie, total sleep time [TST_*ij*_]) into 2 components. The within-person predictor represented the difference between person *j*’s mean and the value on day *i*, such that negative within-person scores indicated lower sleep duration on that day relative to an individual’s average across time. The between-person predictor was the mean for person *j* across all available days such that negative between-person scores indicated lower sleep duration across time for a given person relative to average sleep for all participants in the sample across the study period. Daily mixed-effects models included all participants because these analyses evaluate within-person fluctuations in affect (each adolescent serving as their own control), while accounting for study condition at level 2. Thus, nightly changes in sleep were interpreted independent of whether sleep was experimentally manipulated.

We initially conducted adjusted models of affect as a function of between- and within-person sleep duration (model 1). Next, we conducted adjusted models for morning and evening PA and NA, examining the fixed effect of the experimental stage, condition, and the interaction between the experimental stage and condition to determine whether there was a change in affect (model 2). Next, we examined the effect of the experimental stage, condition, the interaction between the experimental stage and condition, and between- and within-person sleep duration on each of the affect outcomes (model 3). For model 3, at level 1, the daily affect for day *i* and person *j* was modeled by incorporating a person-specific intercept term (*β*_0__*j*_), daily sleep duration (*β*_1_; dTST_*ij*_), and a residual term (*ε_ij_*). At level 2, the person-specific intercept was modeled as a function of person-average sleep duration (*γ*_01_; mTST_*j*_), experimental stage (pre vs post), condition, the interaction between experimental stage and condition, and a random person-specific error term (*υ*_0__*j*_). The daily covariate of exercise (0 = no exercise, 1 = exercise) was added to the models predicting evening PA and the person-level covariate of sex was added to the models predicting morning and evening NA.

## Results

**Figure 3:**
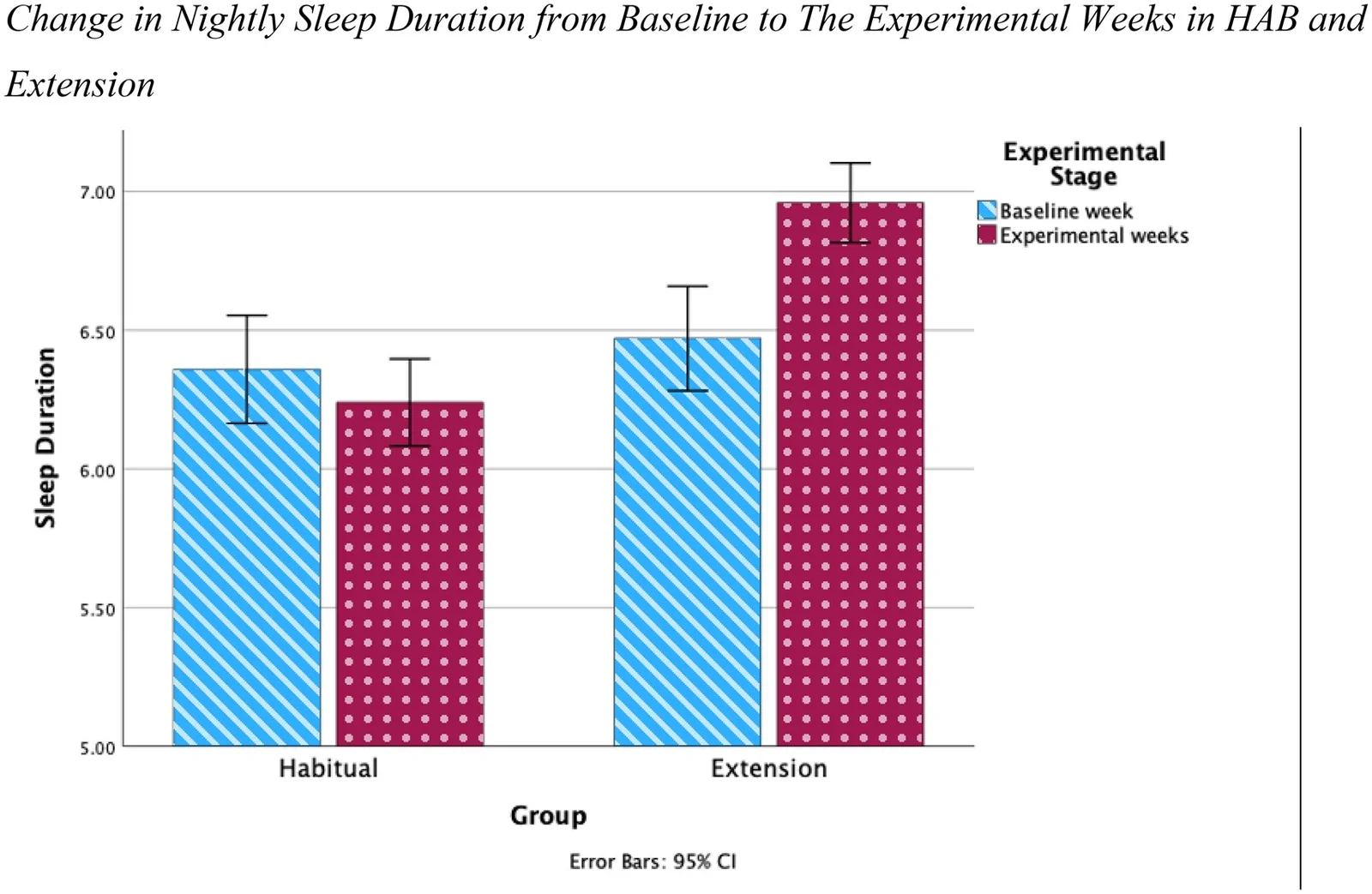
Change in nightly sleep duration from baseline to the experimental weeks in HAB and EXT. Error bars represent 95% CIs. The *y*-axis begins at 5 h to improve visibility of group differences and should be interpreted alongside the reported effect sizes. During the experimental weeks, adolescents in the extension condition slept significantly longer than those in the habitual condition (*P* =.002; Hedges’ *g* =0.89). No significant between-group differences were observed during the baseline week (all *P*-values greater than .15). Abbreviations: EXT, extension; HAB, habitual.

The final sample was comprised of 24 females (58.9%) and 17 males (41.5%); proportions did not significantly differ across the experimental groups (HAB vs EXT; *P*>.05). Participants were healthy adolescents aged 14-17 years who were recruited from local schools and community organizations and met study criteria for short sleep duration. As shown in Table 1, there also were no significant differences between the groups in participant age, sleep characteristics, or affect ratings in week 1. Although small baseline group differences in time in bed and morning NA were observed, these effects were nonsignificant, and our multilevel modeling approach accounts for individual baselines when estimating changes over time. Table 2 provides descriptive statistics for sleep and affect from pre- to postexperimental stage. Figure 3 illustrates the change in nightly sleep duration of the extension group compared to baseline. Adolescents in the EXT group slept significantly longer than those in the HAB group during the experimental weeks (*P* <.05). Consistent with the figure, only the experimental week sleep duration difference between conditions was statistically significant. Notably, 13 out of the 21 adolescents retained in the extension group (62%) adhered to our defined protocol for time in bed manipulation, resulting in an increase of over 30 min in sleep duration.

**Table 1:** Baseline comparison of 2 groups: prerandomization analysis.

	Habitual		Extension		*t* (39)	*P* (2-sided)	Hedges’ *g*
	M	SD	M	SD			
**Time in bed**	7.98	0.96	8.31	0.94	−1.10	0.28	−0.33
**Sleep duration**	6.36	0.70	6.47	0.66	−0.52	0.60	−0.16
**Sleep efficiency**	80.21	6.61	78.88	7.08	0.62	0.54	−0.19
**Sleep quality**	3.32	0.75	3.32	0.71	−0.003	0.99	−0.001
**PA (morning)**	0.72	0.67	0.80	0.69	−0.38	0.70	−0.12
**NA (morning)**	0.21	0.18	0.32	0.35	−1.23	0.23	−0.37
**PA (evening)**	1.25	0.63	1.25	0.77	−0.01	0.99	−0.003
**NA (evening)**	0.55	0.53	0.50	0.50	0.32	0.75	0.10
**Age (years)**	15.29	0.78	15.48	1.03	−0.67	0.50	−0.21

*n* = 41. Values are means (SD) unless otherwise indicated. Hedges’ *g* represents the standardized mean difference between the extension and habitual sleep groups at baseline, with negative signs reflecting slightly higher values in the habitual group. All effect sizes were small (|*g*| < 0.40), indicating no meaningful baseline differences between conditions prior to randomization.

Abbreviations: NA, negative affect; PA, positive affect.

**Table 2:** Sleep characteristics, and affect baseline and postintervention, mean (SD).

	Baseline	Postintervention	Hedges’ *g*
**HAB**			
** Sleep duration (actigraphy-measured)**	6:21 (0:42)	6:13 (0:57)	
** Sleep duration (self-reported)**	7:17 (1:15)	7:19 (1:06)	
** Sleep efficiency**	80.21 (6.61)	77.45 (8.81)	
** Subjective sleep quality**	3.32 (0.75)	3.43 (0.61)	
** Morning PA**	0.72 (0.67)	0.87 (0.65)	
** Morning NA**	0.21 (0.18)	0.25 (0.19)	
** Evening PA**	1.25 (0.63)	1.32 (0.74)	
** Evening NA**	0.55 (0.53)	0.43 (0.25)	
**EXT**			
** Sleep duration (actigraphy-measured)**	6:28 (0:40)	6:58 (0:41)	0.89
** Sleep duration (self-reported)**	7:10 (1:16)	8:30 (1:09)	0.98
** Sleep efficiency**	78.88 (7.08)	74.87 (4.84)	0.20
** Sleep quality**	3.32 (0.71)	3.52 (0.74)	0.14
** Morning PA**	0.80 (0.69)	1.05 (0.86)	0.24
** Morning NA**	0.32 (0.35)	0.37 (0.39)	0.39
** Evening PA**	1.25 (0.77)	1.29 (0.84)	0.04
** Evening NA**	0.50 (0.50)	0.55 (0.54)	0.28

*n* = 41. Values are means (SD). Hedges’ *g* represents the standardized postintervention difference between the extension and habitual sleep groups, with positive values indicating higher scores in the extension condition. Effect sizes are shown only in the extension row because the habitual sleep group serves as the reference group. Standard deviations for sleep duration were converted to decimal hours for consistency.

Abbreviations: EXT, extension; HAB, habitual; NA, negative affect; PA, positive affect.

During the first week, on average participants slept for 6.42 h (SD=1.16) with a corresponding sleep efficiency of 79.56%. During the experimental weeks, individuals in the HAB condition exhibited a mean actigraphy-measured sleep duration of 6.22 h (SD = 0.95) with a sleep efficiency of 77.45% (SD = 8.81). In parallel, participants in the EXT condition had an average actigraphy-measured sleep duration of 6.97 h (SD = 0.68), with a sleep efficiency of 74.87% (SD = 4.84) (see Table 2). The adolescents in the EXT condition experienced a significant increase in sleep duration from week 1 to the experimental weeks (*t*(20)=−0.49, *P *= .002, 95% CI, −0.79 to −0.20). In contrast, no such increase was observed in the HAB sleep condition (*t*(19)=0.14, *P *= .25, 95% CI, −0.10 to 0.38) (Figure 3).

### Main effects of experimental stage and sleep

Despite experimentally increasing sleep duration in the extension group, the primary hypothesis—that increased sleep would be associated with reduced negative affect and increased positive affect—was not supported. No significant differences in morning or evening affect existed between the sleep EXT and HAB sleep conditions across the experimental weeks (all *P*-values greater than .15). We next examined our secondary aim assessing whether day-to-day variation in sleep duration predicted affect irrespective of condition assignment.

#### Morning affect

Positive affect in the morning significantly increased from week 1 to the experimental weeks (*b *= 0.14, SE = 0.07, *P *= .04; Table 3 model 2), while morning NA remained stable (*b *= 0.05, SE = 0.05, *P *= .37; Table 3 model 2). Notably, there were no significant main effects observed for the condition or the interaction between the condition and experimental stage on morning positive or NA.

**Table 3:** Multi-level modeling results for experimental stage, condition, and sleep duration predicting morning and evening affect.

	Morning PA				Morning NA^a^				Evening PA^b^					Evening NA^a^			
	*b*(SE)	*P*	95% CI		*b*(SE)	*P*	95% CI		*b*(SE)	*P*	95% CI		*b*(SE)		*P*	95% CI	
			LL	UL			LL	UL			LL	UL				LL	UL
**Model 1**																	
** Intercept**	0.75 (0.11)	<.001	0.54	0.97	0.18 (0.07)	.01	0.05	0.32	1.18 (0.11)	<.001	0.96	1.39	0.33 (0.09)		.001	0.14	0.52
** Sleep duration (between)**	−0.16 (0.14)	.26	−0.45	0.12	0.07 (0.06)	.20	−0.04	0.19	−**0.31 (0.14)**	**.03**	−**0.59**	−**0.04**	0.09 (0.08)		.29	−0.08	0.25
** Sleep duration (within)**	0.02 (0.01)	.10	−0.01	0.05	−**0.03 (0.01)**	**.006**	−**0.05**	−**0.01**	−0.002 (0.02)	.90	−0.04	0.03	−0.01 (0.01)		.60	−0.03	0.02
**Model 2**																	
** Intercept**	0.73 (0.15)	<.001	0.42	1.04	0.10 (0.08)	.25	−0.07	0.27	1.17 (0.16)	<.001	0.85	1.49	0.37 (0.14)		.01	0.09	0.64
** Experimental stage**	**0.14 (0.07)**	**.04**	**0.01**	**0.28**	0.05 (0.05)	.37	−0.06	0.15	0.05 (0.10)	.59	−0.15	0.26	−0.12 (0.08)		.16	−0.29	0.05
** Condition**	0.08 (0.21)	.71	−0.35	0.51	0.12 (0.09)	.20	−0.06	0.30	0.004 (0.22)	.98	−0.43	0.44	−0.05 (0.15)		.76	−0.36	0.27
Experimental stage * Condition	0.10 (0.09)	.30	−0.09	0.29	0.01 (0.07)	.95	−0.14	0.15	−0.03 (0.14)	.86	−0.41	0.26	0.16 (0.12)		.16	−0.07	0.40
**Model 3**																	
** Intercept**	0.67 (0.15)	<.001	0.36	0.98	0.12 (0.09)	.16	−0.05	0.30	1.08 (0.16)	<.001	0.76	1.40	0.40 (0.14)		.007	0.11	0.69
** Experimental stage**	**0.14 (0.07)**	**.04**	**0.004**	**0.27**	0.04 (0.05)	.40	−0.06	0.14	0.05 (0.10)	.59	−0.15	0.25	−0.12 (0.08)		.15	−0.30	0.05
** Condition**	0.20 (0.22)	.36	−0.24	0.66	0.07 (0.09)	.46	−0.12	0.26	0.18 (0.22)	.42	−0.27	0.63	−0.09 (0.16)		.58	−0.42	0.24
Experimental stage * Condition	0.09 (0.09)	.35	−0.10	0.28	0.02 (0.07)	.74	−0.12	0.17	−0.03 (0.14)	.80	−0.32	0.25	0.17 (0.12)		.16	−0.07	0.41
** Sleep duration (between)**	−0.22 (0.15)	.15	−0.52	0.08	0.06 (0.06)	.30	−0.06	0.19	−**0.33 (0.15)**	**.03**	−**0.62**	−**0.03**	0.08 (0.09)		.40	−0.11	0.26
** Sleep duration (within)**	0.02 (0.01)	.20	−0.01	0.05	−**0.03 (0.01)**	**.005**	−**0.05**	−**0.01**	−0.002 (0.02)	.93	−0.04	0.03	−0.01 (0.01)		.66	−0.03	0.02

*n* = 41. “Experimental stage * Condition” denotes the interaction between condition assignment and study stage (baseline vs experimental weeks). The asterisk (*) reflects the statistical interaction term notation and does not indicate significance. Within-person sleep duration reflects nightly deviations from each adolescent’s mean; between-person sleep duration reflects each adolescent’s average sleep duration across the study period. Bold values indicate statistically significant effects (*p* < .05).

Abbreviations: EXT, extension; HAB, habitual; LL, lower limit; NA, negative affect; PA, positive affect; UL, upper limit.

a Adjusted for sex.

b Adjusted for exercise (y/n).

Sleep duration, whether looking at differences between people or within the same person did not show a significant association with morning PA (*P *> .10), but longer within-person sleep duration was linked to lower morning NA (*b* = −0.03, SE* = *0.01, *P *= .006; Table 3 model 1). Moreover, no significant main effect of between-person sleep duration on morning NA was observed (*P *= .20). That is, how long a given person slept any given night impacted their positive (but not negative) affect the next morning, but there was no difference in morning affect (positive or negative) across experimental groups or even across people of varying sleep durations. Table 3 model 3 displays the associations observed when incorporating all variables into the model. Next, for sensitivity analysis, we added school day vs nonschool day (0 = nonschool day, 1 = school day) as a covariate to the model. Despite this addition, the observed associations remained consistent.

#### Evening affect

Evening positive and NA remained stable throughout the study, with no significant changes observed between week 1 and the experimental weeks (Table 3 model 2). Similar to the morning affect, no significant main effects of the condition or interactions between the condition and experimental stage were detected for evening PA or NA (Table 3 model 2).

Longer between-person average sleep duration was associated with lower PA in the evening (*b = *−0.31, SE* = *0.14, *P *= .03), while within-person sleep duration was not associated with PA (*P *= .90) (Table 3 model 1). There were no significant main effects of between- or within-person sleep duration on evening NA (*P *> .29). In other words, adolescents who slept longer differed in evening positive (but not negative) affect, independent of the sleep manipulation or night-to-night changes in sleep, both of which showed no effects on evening affect. Table 3 model 3 displays the associations observed when incorporating all variables into the model. In sensitivity analyses adding school day vs nonschool day as a covariate to the model did not substantively alter findings.

## Discussion

In this study, we employed experimental sleep EXT^20^ to examine the effects of increasing sleep duration on daily affect in adolescents, to evaluate whether extending sleep duration would be associated with changes in daily affect. Healthy adolescents averaging less than the recommended 8 h per night completed 1 week of baseline actigraphy, then were randomized for 2 weeks to continue HAB short sleep or extend time in bed by 90 min (EXT). As intended, adolescents in EXT averaged longer sleep than their baseline and compared to HAB. However, these gains did not translate to improvements in affect relative to the HAB sleep group, as reflected by the small effect sizes, contrary to our primary hypothesis.

Adolescents in both HAB and EXT reported higher morning PA in the experimental weeks compared to baseline. These within-person and between-person sleep-affect findings reflect our planned secondary aim and should be interpreted with caution in light of the null experimental condition effects. Although study week did not impact morning NA on average, on nights when participants slept longer, they reported lower morning NA. Evening results were contrary to hypotheses: adolescents who slept longer on average tended to report lower PA in the evening, and neither experimental manipulation nor nightly variability impacted evening affect.

This sleep EXT study confirmed that, for many short-sleeping adolescents, a brief problem-solving session can lengthen sleep over a two-week span. This is particularly noteworthy considering the widespread concern that at least half of adolescents do not meet sleep recommendations.^4^ While our results align with a previous study that informed the design of our research, it is noteworthy that the magnitude of change was more substantial in that study (1 h and 18 min)^20^ compared to the present study (35 min). This discrepancy may partially reflect differences in how sleep duration was measured across the 2 studies or variations in study design.^20^ Van Dyk et al^20^ used a counterbalanced crossover design in which adolescents experienced both HAB sleep and sleep EXT over 2-week periods, with actigraphy and diaries reviewed throughout the intervention to reinforce adherence. In contrast, the present study used a parallel-group randomized design in which daily diaries were reviewed in real time, whereas actigraphy data were scored only after the study period. Although adolescents in the extension condition were instructed to increase time in bed by 90 min, they obtained less than half that amount as additional sleep, highlighting the challenge of advancing bedtime on school nights. Even so, participants achieved a modest but reliable increase in sleep duration, supporting the feasibility of targeting sleep among short-sleeping adolescents. Despite this difference, the consistent improvement underscores the feasibility of targeting sleep duration in adolescents.

Participants reported a significant increase in morning PA from the baseline to the experimental phase of the study, a pattern that was similar for both the HAB and EXT conditions. One possible explanation for this change could be the decrease in sleep variability during the experimental weeks, resulting from fixed bedtime and wake-up times to minimize sleep fluctuations. There is some evidence that less night-to-night variation in sleep patterns is associated with less negative mood, but findings have been inconsistent and are largely correlational.^32^ Another potential contributing factor is the impact of monitoring itself, as research suggests that behaviors such as sleep, weight, and nutrition can improve simply through the process of being monitored (eg, Burke et al^33^). The present study design did not include a control group whose sleep variability was not manipulated, which would help to address these possibilities.

Increased PA may protect against emerging psychological challenges during this formative life stage. It plays a foundational role in mental health, influencing psychological and physiological processes,^34^ buffering stress through adaptive coping, and enhancing psychological flexibility.^35^ Positive affect is linked to neurotransmitters such as dopamine and serotonin, critical for mood regulation and resilience,^36^ and supports cardiovascular health and reduced inflammation.^36^ Socially, higher PA fosters prosocial behavior and stronger interpersonal connections, building support networks that enhance resilience.^37^ Positive affect is both a marker of well-being and a mechanism that promotes recovery from adversity and promotes mental health.^38^^,^^39^

Our findings also suggest a protective role of longer daily sleep duration in reducing NA among short-sleeping adolescents. While we found no reliable effect of experimental stage or condition (HAB vs EXT) on NA, individuals reported lower morning NA after nights of longer sleep. This builds on prior work linking sleep and NA, extending it by showing a within-person effect using objective, actigraphy-measured sleep—unlike Shen et al,^40^ which found between-person effects and associations only with self-reported sleep. This supports a dynamic, day-to-day relationship between longer sleep and lower NA. While Talbot et al^13^ examined acute deprivation, our results show even modest sleep EXT may benefit mood within an individual, though the lack of a condition effect limits causal interpretations. These effects may partly reflect extended sleep’s role in regulating mood-related neurotransmitters such as serotonin and dopamine.^41^

Our design allowed assessment of how changes in sleep duration among short-sleeping adolescents impact NA within the same individual over time. Intensive longitudinal designs using both short- and nonshort-sleeping adolescents can be used in subsequent studies to identify distinctions and explore underlying mechanisms. Investigating individual differences and moderating factors such as stress or lifestyle variables (eg, social interactions, daily activities)^42^^,^^43^ could further clarify this relationship. For example, Bai et al^42^ found adolescents slept 26 min longer after days with positive parent interactions, and another study linked daily family interactions to evening NA, particularly among females.^44^ Examining such factors may deepen understanding of the relationship between sleep and NA.

The negative relationship between between-person sleep duration and evening PA suggests that, on average, individuals with longer overall sleep experienced lower evening PA. Although hypersomnia has been linked to mood disturbance,^45^ our sample was limited to short-sleeping adolescents (mean = 6.53 h, SD = 1.27; maximum = 12.07 h). In this context, “longer overall sleep” refers to adolescents who obtained relatively more sleep within this short-sleeping sample, approximately +1 SD above the mean, or close to 8 h. This association may reflect individual differences in sleep quality or architecture among these adolescents. Disruptions in continuity, such as increased awakenings or changes in REM and deep sleep, could contribute to diminished evening PA. Further research examining sleep patterns among adolescents who obtain typical or recommended sleep durations (rather than short sleepers) may help clarify this relationship.

The increase in PA and the link between daily sleep variability and NA were specific to morning affect, suggesting sleep’s influence may be time-dependent. Morning affect may be more responsive to the prior night’s sleep,^46^ while evening affect, although assessed as “current” affect, may reflect the day’s experiences, stressors, and emotional state following coping efforts.^47^ The inverse association between between-person sleep duration and PA was specific to evening, raising the possibility that more sleep serves as a coping mechanism for those prone to worse evening affect. Individual differences, such as chronotype and personality traits, may also shape these patterns.^48^ Parsing these factors will be important for understanding diurnal variations in emotional well-being and their implications for mental health.

There are several possible explanations for why increased sleep duration did not lead to better affect in the extension group. First, the manipulation produced modest gains in actual sleep (∼35 min), and adolescents still slept below recommendations (average ∼7 h). Second, the intervention lasted only 2 weeks, providing limited time for behavioral adaptation or accumulation of meaningful sleep-related benefits. Because affect was assessed concurrently during this brief period, any emotional effects of improved sleep may have been too small or too early to detect. Third, although adolescents were instructed to add 90 min in bed, they slept far less of that additional time, which may have reduced sleep efficiency and impacted emotional outcomes. Fourth, the use of fixed bed- and rise-times in both conditions intentionally reduced sleep variability, which may have influenced affect through pathways unrelated to sleep duration alone. Fifth, selective enrollment may have contributed to attenuated effects, as adolescents who volunteered for a sleep intervention study may differ systematically from short-sleeping adolescents in the general population in motivation, sleep habits, or affective functioning. Sixth, the protocol primarily relied on an “early-to-bed” approach to extending sleep, which may not align with the circadian timing of short-sleeping adolescents with later chronotypes. Prior work suggests that night-oriented adolescents may have more difficulty extending sleep using earlier bedtimes and may derive fewer affective benefits from this approach compared to morning-oriented adolescents.^49^ Finally, our sample size was relatively small, which may have limited power to detect subtle and time-dependent affective responses.

It is important to interpret these findings in light of several limitations. First, the study was designed to isolate and manipulate sleep duration, with variability intentionally minimized in both conditions through consistent bedtimes and rise-times. While this increased internal control, it may have constrained the ability to detect effects driven by natural fluctuations in sleep timing. Second, the sample included only healthy adolescents with less-than-recommended sleep, and although participants were broadly representative of the local community, the modest sample size limits the ability to examine how sleep and affect associations differ across sociodemographic groups. The small sample also reduces power to detect smaller effects. Monitoring itself may have influenced sleep or affect, as self-tracking can alter behavior. In addition, within-person reliability was lower for NA than for PA (findings not shown), which may have reduced sensitivity to NA changes. Further, although our affect measures included both high- and low-arousal items, the current analyses focused on composite PA and NA scores to reduce the number of tests and maintain statistical power. Future studies with larger samples could examine whether specific affective dimensions (eg, low-arousal PA, depressed vs anxious NA) are differentially sensitive to changes in sleep duration. Next, the brief 2-week intervention precludes assessment of long-term or sustained affective changes. Additionally, the mixed-effects models that combined participants across both conditions to evaluate nightly sleep-affect associations should be interpreted with caution. Although they account for condition at the within-person level, these analyses are conceptually secondary to the randomized comparison and cannot isolate effects driven uniquely by the experimental manipulation. Finally, the POMS vigor items are well supported in daily diary research, but the adapted PA scale we used has not yet been independently validated.

In summary, this study shows that extending sleep among short-sleeping adolescents is feasible over a short period of time. However, the experimental manipulation did not lead to improvements in affect relative to the HAB sleep group, suggesting that more substantial increases in sleep or longer interventions may be needed to observe affective changes. The daily sleep-affect associations we observed, such as longer within-person sleep predicting lower morning NA and longer between-person sleep predicting lower evening PA, should be interpreted with caution, as they were not driven by differences between the randomized conditions. Additional work is needed to identify intervention components or contextual factors that could more reliably influence both sleep and emotional well-being in adolescence.

## Data Availability

The datasets generated and/or analyzed during the current study are available from the corresponding author on reasonable request and with appropriate institutional approvals.

## Open science statement

Data and analytic code are available from the corresponding author upon reasonable request. This study was not preregistered.
